# Suppressor of Cytokine Signaling (SOCS) 5 Utilises Distinct Domains for Regulation of JAK1 and Interaction with the Adaptor Protein Shc-1

**DOI:** 10.1371/journal.pone.0070536

**Published:** 2013-08-21

**Authors:** Edmond M. Linossi, Indu R. Chandrashekaran, Tatiana B. Kolesnik, James M. Murphy, Andrew I. Webb, Tracy A. Willson, Lukasz Kedzierski, Alex N. Bullock, Jeffrey J. Babon, Raymond S. Norton, Nicos A. Nicola, Sandra E. Nicholson

**Affiliations:** 1 Walter and Eliza Hall Institute of Medical Research, Parkville, Victoria, Australia; 2 The University of Melbourne, Parkville, Victoria, Australia; 3 Monash Institute of Pharmaceutical Sciences, Monash University, Parkville, Victoria, Australia; 4 Structural Genomics Consortium, University of Oxford, Oxford, United Kingdom; University of Dundee, United Kingdom

## Abstract

Suppressor of Cytokine Signaling (SOCS)5 is thought to act as a tumour suppressor through negative regulation of JAK/STAT and epidermal growth factor (EGF) signaling. However, the mechanism/s by which SOCS5 acts on these two distinct pathways is unclear. We show for the first time that SOCS5 can interact directly with JAK via a unique, conserved region in its N-terminus, which we have termed the JAK interaction region (JIR). Co-expression of SOCS5 was able to specifically reduce JAK1 and JAK2 (but not JAK3 or TYK2) autophosphorylation and this function required both the conserved JIR and additional sequences within the long SOCS5 N-terminal region. We further demonstrate that SOCS5 can directly inhibit JAK1 kinase activity, although its mechanism of action appears distinct from that of SOCS1 and SOCS3. In addition, we identify phosphoTyr317 in Shc-1 as a high-affinity substrate for the SOCS5-SH2 domain and suggest that SOCS5 may negatively regulate EGF and growth factor-driven Shc-1 signaling by binding to this site. These findings suggest that different domains in SOCS5 contribute to two distinct mechanisms for regulation of cytokine and growth factor signaling.

## Introduction

Enhanced survival, proliferation, angiogenesis and/or migration are hallmarks of many human cancers [1]. Frequently, the increased expression and activation of protein tyrosine and serine/threonine kinases are important events in neoplastic transformation and disease progression. For example, activating forms of the EGF receptor (EGF-R) are prevalent in cancers such as glioblastoma, head and neck cancers, small cell lung carcinomas and breast and colon cancers [2], [3]. Similarly, activating mutations in JAK are associated with various myeloproliferative and lymphocytic leukemias [4]–[6]. Previous studies have suggested that SOCS5 can regulate both EGF-R and JAK signaling in mammalian cells [7]–[10], and the *Drosophila* homologue of SOCS5 (SOCS36E) has been shown to regulate both JAK/STAT and EGF receptor signaling *in vivo* [11], [12], implying a conserved ancestral function. Here we provide a molecular explanation as to how these two distinct SOCS5 activities might be mediated, and hence how SOCS5 might impact on these cancer-promoting kinase cascades.

The Janus kinases (JAKs) sit at the apex of many cytokine receptor pathways and their activation results in phosphorylation of the cytoplasmic domains of the receptor, leading to the recruitment and phosphorylation of the Signal Transducers and Activators of Transcription (STAT)s. In turn, the STATs induce transcription of a specific subset of genes, resulting in an appropriate cellular response that can include survival, proliferation and/or cell differentiation. However, this cellular response requires tight regulation, as aberrant signaling has been unequivocally linked to mutations in key signaling genes, such as the valine 617 mutation in the JAK2 pseudokinase (JH2) domain associated with myeloproliferative disease [6], and the JAK1- and JAK2-activating mutations associated with acute lymphoblastic leukemia (ALL) [4], [5]. Similarly, mutations in the IL-7 α-receptor, which result in constitutive activation of JAK1, are associated with a sub-group of T cell ALL patients [13]. Since their discovery in the late nineties [14]–[16], the Suppressor of Cytokine Signaling (SOCS) proteins are now recognised as one of the most critical cellular mechanisms for controlling cytokine responses [17]. The SOCS proteins are also transcriptionally regulated by the STATs and by a variety of mechanisms, serve to inhibit JAK signaling in a classic negative feedback loop.

The eight mammalian SOCS proteins, SOCS1-7 and cytokine inducible SH2 domain-containing protein (CIS) consist of a C-terminal SOCS box, a central SH2 domain and an N-terminal region of variable sequence and length [15], [18]. Mechanistically, the highly conserved SOCS box motif forms part of an E3 ubiquitin ligase complex, consisting of elongins B and C, Cullin5 and Rbx2, which mediates the ubiquitination and proteasomal degradation of SH2-bound substrates [19]. SOCS2 and CIS can also bind, via their SH2 domains, to tyrosine phosphorylated sites within receptor cytoplasmic domains, and may compete with and block access of STAT molecules and consequently block further STAT activation [20]–[23].

SOCS1 and SOCS3, which appear to have a unique ability to inhibit JAK catalytic activity, contain a Kinase Inhibitory Region (KIR) adjacent to the SH2 domain that is critical for their inhibition of JAK activity [24], [25]. The mechanism by which SOCS3 interacts with and inhibits JAK has been described recently, whereby the SH2 domain binds a phosphotyrosyl residue within the IL-6 signaling receptor, gp130, and together with the KIR region, simultaneously binds and inhibits the JAK catalytic (JH1) domain [26], [27]. This tripartite binding between JAK/receptor/SOCS3 results in a highly specialised, specific and potent inhibition of JAK-mediated signal transduction. Interestingly, SOCS3 can inhibit JAK1, JAK2 and TYK2, but not JAK3 [26], providing further specificity in the regulation of the JAK/STAT system. Equivalent detail is currently lacking for SOCS1, which has been reported to bind phosphotyrosines in both the JAK activation loop and the interferon (IFN) receptor cytoplasmic domains [24], [28], [29].

SOCS4, 5, 6 and 7 are distinguished from other SOCS proteins by an extended N-terminal region, which varies from 270 to 385 amino acid residues in length for the mouse proteins [18]. These long SOCS N-termini are predicted to be disordered [30] and share no sequence homology with protein domains in existing databases. Although little information is available regarding their function, they are predicted to mediate protein interactions [30]. This has certainly been demonstrated for SOCS6, which requires its N-terminal region to interact with the active form of the T cell specific kinase Lck [31]. Most recently, a highly conserved ∼70-residue region was identified in the N-termini of SOCS4 and SOCS5, indicating a potential role for this region in the function of both proteins [30].

Whilst the physiological functions of SOCS1-3, and to a lesser extent those of CIS, SOCS6 and SOCS7 have been described, the biological roles of SOCS4 and SOCS5 remain poorly characterised. Currently, SOCS5 is thought to negatively regulate interleukin (IL)-4 signaling, polarizing CD4^+^ T cells towards a Th1 phenotype and has been suggested to bind the IL-4 receptor (IL-4R) α chain via the first 100 residues of its N-terminal region, displacing JAK1 from the receptor complex to inhibit further signaling [32]. Paradoxically, however, mice deficient in SOCS5 do not appear to have defects in IL-4 signaling and have been shown to mount a normal Th2-mediated response to the intracellular parasite *Leishmania major* [33]. Thus, the physiological role of SOCS5 is yet to be elucidated.

Growing evidence now points towards a role for SOCS5 as a tumor suppressor. Early studies utilising exogenous expression of SOCS5 suggested a role in inhibition of EGF signaling, with SOCS5 shown to interact with the EGF-R complex in a ligand-independent manner [7], [8]. SOCS5-deficient mice develop normally [33], implying that SOCS5 is unlikely to regulate EGF-R signaling in the context of embryonic development. However, it remains possible that SOCS5 may act redundantly with other SOCS family proteins, particularly given the 92% amino acid sequence identity shared between the SOCS4 and SOCS5-SH2 domains. More recently, epigenetic silencing of SOCS5 expression has been shown to correlate inversely with EGF-R expression in aggressive hepatocarcinoma [9], while down-regulation of SOCS5 expression by tumor-derived miR-9 results in enhanced JAK1/2 and STAT1/3 phosphorylation in endothelial cells [10]. In the latter study, inhibition of miR-9 resulted in reduced cell migration and reduced tumor burden in mice; however, although SOCS5 was identified as a target of miR-9, the mechanism by which increased levels of SOCS5 inhibited JAK activity was not elucidated [10].

The EGF-R and JAK are both validated targets for the treatment of human cancer, with inhibitors in use in the clinic and in phase III clinical trials [2], [34], [35]. Here we identify a previously uncharacterised region in the extended SOCS5 N-terminus that can bind directly to the JAK kinase domain. We also present evidence that SOCS5 can impact on JAK1 and JAK2 activation and has the capacity to act as a direct kinase inhibitor. In addition, we identify a novel target for the SOCS5-SH2 domain, Tyr317 in Shc-1, and propose that SOCS5 could act to regulate EGF-R-Shc-1-Grb2 signaling. Our studies indicate that SOCS5 is likely to utilise different domains and multiple interaction points to regulate both JAK and EGF-R signaling. This work will help address the potential regulatory function of SOCS5 in the context of oncogenic signaling.

## Materials and Methods

### Mammalian expression vectors

The cDNA encoding SOCS5 has been described previously [18]. Constructs encoding SOCS5 with an N-terminal Flag epitope tag (DYKDDDDK) were generated by polymerase chain reaction (PCR) to give fragments with in-frame *Asc* I and *Mlu* I restriction sites at the N- and C- termini respectively and sub-cloned into the mammalian expression vector pEF-FLAG-I, a derivative of the mammalian expression vector pEF-BOS [36]. SOCS5 deletion mutants lacking either the full N-terminus (residues 370 to 536; Δ369), or with various N-terminal truncations (Δ 110, Δ 171, Δ 313 and Δ 349) were generated by PCR. The SOCS-5 SH2 mutant in which the invariant arginine was replaced by lysine (R406K; mSH2), mutation of the putative “KIR” region (H360A), mutations in the SOCS5 SOCS box to eliminate elongin C binding (L484P, C488F; mSB) and deletion of the conserved N-terminal fragment (Δ 175–244), were generated using the PCR-based technique, splicing by overlap extension [37]. Mouse JAK1, JAK2 and TYK2, and human JAK3 sequences were sub-cloned into the mammalian expression vector pEF-FLAG-I to give proteins with an N-terminal Flag epitope. The cDNA encoding Flag epitope-tagged Shc-1 was cloned into a pCAGs vector and expresses a 2Flag-GFP-Shc-1 fusion protein (kindly provided by the Pawson laboratory; MSHRI, Toronto).

### Expression and purification of recombinant proteins

#### SOCS5^175–244^

The fragment in the N-terminus of mouse SOCS5 (residues 175–244), corresponding to the region conserved in SOCS4, was amplified from SOCS5 cDNA and engineered to contain a Tobacco Etch Virus (TEV) protease cleavage site upstream of the SOCS5^175–244^ sequence. The construct was ligated into the pGEX-2T vector (GE Healthcare) via EcoRI sites and transformed into *E*. *coli* BL21 (DE3) cells. SOCS5^175–244^ was expressed as a fusion protein with a glutathione S-transferase (GST) tag in 1 L of Luria-Bertani medium. The cells were grown to an OD_600_ 0.8 at 28°C, cooled to 18°C and protein expression was induced with 1 mM isopropyl β-D-1-thiogalactopyranoside (IPTG) for 20 h at 18°C. The fusion protein, expressed as a soluble protein, was purified using glutathione-Sepharose^TM^ 4B (GE Healthcare) according to the manufacturer's instructions. One unit of TEV per 20 mg of fusion protein was used to cleave at 4°C for 20 h on a rotating mixer. The polypeptide corresponding to SOCS5^175–244^ was purified from the cleavage mixture by RP-HPLC (Phenomenex; 50 mm×21.20 mm C8 column, 100 Å pore size) using a gradient of 20% to 60% acetonitrile and 0.1% trifluoroacetic acid over 20 min. The purity of SOCS5^175–244^ was confirmed by analytical RP-HPLC and the molecular mass determined by LC-MS (8103 Da).

#### SOCS5-SH2 domain

Recombinant SOCS5-SH2 domain was engineered to contain an N-terminal GST-tag and included the SOCS box sequences for increased stability and solubility when expressed as a ternary complex with elongins B and C, as previously described [38]. *E. coli* expression vectors encoding human SOCS5 (residues 358–529; vector PGTVL2) and elongin B/elongin C (residues 1–118 and 17–112 respectively; vector pACYCDUET) were co-transformed into BL21(DE3) cells for expression and purification of the trimeric SOCS5-SH2-SOCS box-elongin B/elongin C complex (GST-SOCS5-SH2 Elo B/C). Cells were grown to an O.D. of 0.8 at 37°C, cooled and protein expression induced with 1 mM IPTG for 12–16 h at 18°C. Cells were collected by centrifugation and lysed in phosphate buffered saline (PBS) containing 0.5 mM tris(2-carboxyethyl)phosphine (TCEP), 1 mM phenylmethylsulfonyl fluoride (PMSF; Sigma) and 0.005% (w/v) hen egg white lysozyme (Sigma) by sonication in a Sonoplus sonicator (BANDELIN). Affinity purification was performed using gravity filtration with glutathione-Sepharose^TM^ 4B according to the manufacturer's instructions. Recombinant GST-SOCS5-SH2 Elo B/C was further purified by size exclusion chromatography with a HiLoad^TM^Superdex^TM^ 200 (16/60) column (Pharmacia Biotech) at 1.0 mL/min flow rate in 20 mM Tris-HCl, pH 7.5, 150 mM NaCl, 0.5 mM TCEP.

#### SOCS3-SH2 domain

The construct for expression of recombinant murine SOCS3 lacks the first 21 amino acids and has the PEST motif (residues 129–163) replaced by a Gly-Ser x4 linker, as these modifications enhance its stability and solubility. SOCS3 protein was expressed and purified as described [39], [40].

#### JAK JH1 domains

Recombinant JAK JH1 domains were expressed in insect cells and purified essentially as described [41]–[43].

#### Src kinase domain

was kindly provided by Dr. Nadia Kershaw (Walter & Eliza Hall Institute) and was expressed and purified essentially as described [44].

### Antibodies

Anti-phosphoJAK1 antibody was obtained from Biosource. Antibodies to phosphoJAK2 were obtained from Cell Signaling. The pan-anti-phosphotyrosine antibody (4G10) was obtained from Millipore. Rat anti-Flag antibody was a kind gift from Prof. D. Huang & Dr. L. O'Reilly, (Walter and Eliza Hall Institute). The anti-SOCS5 antibody was generated in-house and is a mouse monoclonal antibody directed against the SOCS5 N-terminal region.

### Transient transfection of 293T cells

293T cells [45] were maintained in DMEM supplemented with 100 U/mL penicillin, 0.1 mg/mL streptomycin and 10% fetal bovine serum (Sigma). Cells were transiently transfected using FuGene6 or FuGeneHD (Promega) according to the manufacturer's instructions.

### Luciferase Assay

293T cells were transiently transfected with 500 ng of an IL-4-responsive promoter-*Firefly* luciferase reporter gene [p(Ie-IL4RE)_4_-luc] [46], 5 ng hStat6 DNA, and constructs encoding Flag epitope-tagged SOCS proteins. To control for transfection efficiency, cells were co-transfected with 20 ng of a vector expressing *Renilla* luciferase downstream of the Herpes-simplex virus thymidine kinase promoter (HSV-TK) (Promega). Cells were incubated overnight with or without 10 ng/mL recombinant human IL-4 (R&D systems) prior to lysis with 100 μL of Reporter Lysis Buffer (Promega) containing protease inhibitors (Complete Cocktail tablets, Boehringer Mannheim). *Firefly* and *Renilla* luciferase activities were quantified using substrate reagents from the Luciferase Assay Dual-Reporter kit (Promega) and an automated LUMIstar Galaxy plate reader (BMG Technologies). SOCS expression was analyzed by Western blotting with polyclonal rat anti-Flag antibody.

### Immunoprecipitation and Western Blot

Cells were lysed in KALB lysis buffer [47] containing protease inhibitors (Complete Cocktail tablets, Roche), 1 mM PMSF, 1 mM Na_3_VO_4_ and 1 mM NaF. Proteins were immunoprecipitated using anti-Flag antibody conjugated to Sepharose (M2; EASTMAN KODAK). Proteins were separated by sodium dodecyl sulphate-polyacrylamide gel electrophoresis (SDS-PAGE) under reducing conditions and electrophoretically transferred to Biotrace polyvinylidene fluoride (Pall Corp.) or nitrocellulose membranes (Amersham). Membranes were blocked overnight in 10% w/v skim milk and incubated with primary antibody for 2 h. Antibody binding was visualized with peroxidase-conjugated goat anti-rat immunoglobulin (Southern Biotech), sheep anti-rabbit immunoglobulin (Chemicon), sheep anti-mouse immunoglobulin (GE Healthcare), or goat anti-mouse immunoglobulin, light chain specific (Jackson ImmunoResearch) and the enhanced chemiluminescence (ECL) system (Amersham or Millipore). To re-blot, the membranes were stripped of antibodies in 0.1 M glycine, pH 2.9.

#### SOCS5: Shc-1 co-immunoprecipitation

Cells were pre-treated with 10 μM MG132 (Sigma) for 3 h followed by treatment with pervanadate solution (H_2_O_2_/25 μM Na_3_VO_4_) for 30 min and lysis in 1% NP-40 buffer (1% v/v NP-40, 50 mM HEPES, pH 7.4, 150 mM NaCl, 1 mM EDTA, 1 mM NaF, 1 mM Na_3_VO_4_). Cell lysates were pre-cleared with protein-A-Sepharose for 1.5 h prior to immunoprecipitation of Flag-tagged Shc-1 proteins with anti-Flag M2 affinity gel (Sigma). Co-immunoprecipitated SOCS5 protein was detected by Western blotting with in-house anti-SOCS5 antibodies.

### In vitro kinase inhibition assays

#### Autophosphorylation

JAK1 *in vitro* kinase assays were performed essentially as described [48] with the inclusion of 1 mM dithiothreitol in the kinase reaction. Proteins were then separated by 10% acrylamide SDS-PAGE and electrophoretically transferred to PVDF membrane. Incorporation of [γ-^32^P]-ATP was analyzed using a PhosphorImager (Fujifilm FLA-3000).

#### Substrate phosphorylation

Constructs encoding full-length, Flag-tagged JAK1, SOCS1, SOCS3 and SOCS5 were independently transfected into 293T cells, and cells were lysed as described for immunoprecipitation and Western blotting. Proteins were then immunoprecipitated with anti-Flag antibody and eluted from the M2 Sepharose resin using 5× bead volumes of Flag peptide (Sigma Aldrich) at a concentration of 100 µg/mL in kinase assay buffer (20 mM HEPES, pH 7.5, 200 mM NaCl, 2 mM MnCl_2_, 2 mM MgCl_2_ and 2 mM DTT). The eluted proteins were then concentrated, mixed appropriately and incubated with 10 mM ATP and 1 mg/mL GST-Jak2 activation peptide as a substrate [49] for 15 min at 37°C. Proteins were then separated by SDS-PAGE and electrophoretically transferred to nitrocellulose membrane. Incorporation of ATP was detected using phosphospecific antibodies. Protein levels were determined by Western blotting with anti-Flag antibodies.

#### JIR (SOCS5^175–244^)

GST-JAK2 substrate peptide (1 mg/mL) was incubated with 50 nM JAK1 JH1 at 25°C for 15 min in kinase assay buffer together with 1 mM ATP and various concentrations of either recombinant SOCS3 or SOCS5^175–244^. 1 mCi [γ-^32^P]-ATP was included to allow visualization of phosphorylation via autoradiography and phosphorimaging. After incubation, the reactions were boiled and subjected to analysis by SDS-PAGE. Gels were first stained with Simply Blue^TM^ (Invitrogen) to visualize proteins and were subsequently analyzed using a PhosphorImager.

### Surface Plasmon Resonance (SPR)

All SPR experiments were conducted on a BIAcore3000 instrument.

#### Analysis of SOCS^175–244^ binding to JAK JH1 domains

Recombinant SOCS5^175–244^ was diluted to 10 μg/mL in 10 mM sodium acetate, pH 4.5 and immobilised by amine coupling to a CM5 Biosensor chip (GE Healthcare) according to the manufacturer's specifications. A reference flow cell was prepared by the same procedure in the absence of protein. Increasing concentrations (62.5 nM to 2 µM) of recombinant JAK JH1 (kinase) domain were injected at a rate of 20 µL/min for 2 min in running buffer (10 mM HEPES, pH 8.0, 200 mM NaCl, 3.0 mM EDTA, 0.05% v/v Tween20). The surface was regenerated between injections with 50 mM NaOH at a rate of 20 µL/min for 20 s. The reference flow cell sensorgrams were subtracted from the ligand flow cell sensorgrams for all analyses. Saturation curves were obtained by plotting the response signal at equilibrium as a function of the analyte concentration. These curves were fitted to a steady-state model to derive the apparent equilibrium dissociation constant (*K*_D_). *K*_D_ values were representative of three (JAK1) and one (JAK3 and TYK2) experiments. GraphPad Prism software (version 6.0; GraphPad software, Inc.) was used for the analysis of steady-state SPR data.

#### Analysis of SOCS5-SH2 domain binding to phosphopeptides

The binding affinities of the SOCS5-SH2 domain for different tyrosine phosphorylated peptides were determined by a competitive binding assay. Shc-1 pY317 was immobilised by amine coupling to a CM5 Biosensor Chip (GE Healthcare) according to the manufacturer's instructions. Recombinant GST-SOCS5-SH2 Elo B/C was then mixed at 0.1 µM with serially diluted competitor peptides (41 nM to 10 µM) in running buffer (20 mM HEPES, pH 7.4, 150 mM NaCl, 3.4 mM EDTA, 0.05% v/v Tween20) and injected onto the chip at a flow rate of 20 µL/min, for 2 min. Non-specific binding of the recombinant protein to a reference lane on the chip was subtracted within the experiment and regeneration was achieved with 10 mM glycine-HCL, pH 2.0. The binding affinities of the competitor peptides were determined by steady-state analysis as follows. The measured response of SOCS5 binding in the presence of competitor was expressed as a percentage of total binding (SOCS5 binding to immobilised Shc-1 pY317 peptide in the absence of competitor). A plot of the log_10_ competitor peptide concentration versus percentage of total binding was then used to fit a non-linear regression and an equilibrium dissociation constant determined. *K*_D_ values are representative of two replicate experiments. All phosphopeptides were purchased from GL Biochem, China.

## Results

### SOCS1 and SOCS5 are unique in their ability to inhibit JAK1 activation

**Figure 1:**
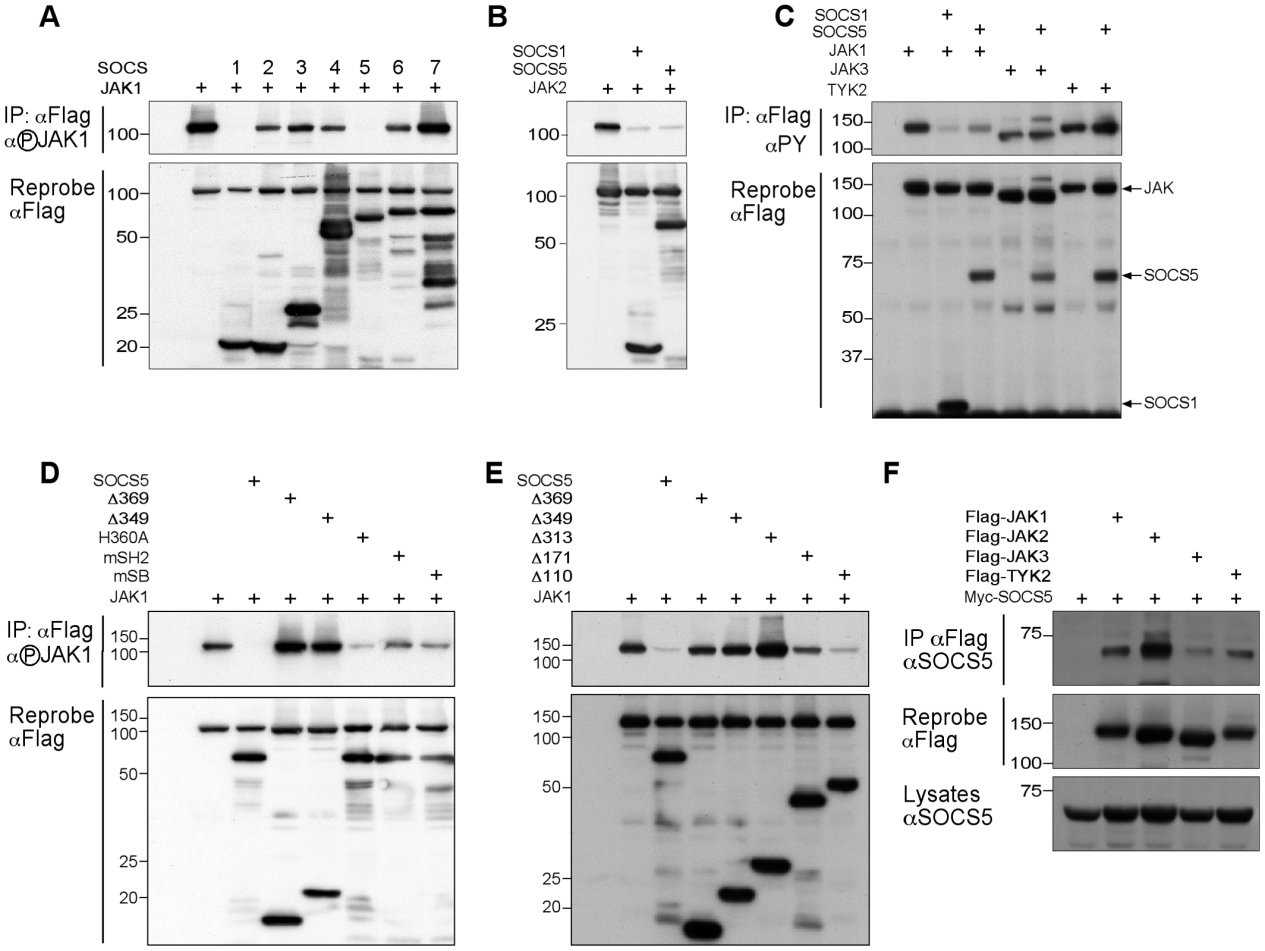
SOCS5 can specifically block JAK1 and JAK2 autophosphorylation and the SOCS5 N-terminus is critical for inhibition of JAK. (A) 293T cells were transfected with cDNA encoding Flag-tagged mouse JAK1 (+) in the presence or absence of cDNAs encoding Flag-tagged SOCS1-SOCS7. 293T cells were transfected with cDNA encoding (B) Flag-tagged JAK2, (C) JAK3 or TYK2 in the presence of either SOCS1 or SOCS5. (D & E) 293T cells were transfected with cDNA encoding Flag-tagged mouse JAK1 (+) in the presence or absence of cDNAs encoding Flag-tagged SOCS5 or various SOCS5 mutants with either N-terminal truncations (Δ369, Δ349, Δ313, Δ171, Δ110) or with His360 (H360A), the SH2 domain (mSH2) or SOCS box (mSB) mutated. (A–E) Cells were lysed and anti-Flag immunoprecipitates analyzed by Western blot with phospho-specific (JAK1: A, D & E; JAK2: B) or anti-phosphotyrosine antibodies (αPY) (JAK1, JAK3 & TYK2; C) (upper panels). The blots were stripped and reprobed with rat anti-Flag antibody (lower panels). (F) 293T cells were transfected with cDNA encoding Myc-tagged SOCS5 (+) in the presence or absence of cDNA encoding Flag-tagged JAK1, JAK2, JAK3 or TYK2. Cells were lysed and anti-Flag immunoprecipitates analyzed by Western blot with anti-SOCS5 antibodies (top panel). The blot was stripped and reprobed with anti-Flag antibodies (middle panel). Cell lysates were blotted with anti-SOCS5 antibodies (bottom panel). Panels A, B, D and E are 10% acrylamide gels. Panels C and F are 4–12% gradient gels.

Given that SOCS1 and SOCS3 have been reported to interact directly with JAK and inhibit catalytic activity [24], [26], [50], we first tested whether SOCS5 could inhibit JAK autophosphorylation when both SOCS5 and JAK were co-expressed. 293T cells were transiently transfected with plasmids encoding Flag-tagged JAK1 with or without Flag-tagged SOCS1 to 7. JAK1 activation was detected by immunoprecipitation with anti-Flag antibodies followed by Western blot with a phospho-specific JAK1 antibody recognizing the critical catalytic loop Tyr1033 and 1034. At high expression levels JAK becomes constitutively active and tyrosine phosphorylated in the absence of cytokine and growth factor stimulation (Fig. 1A, first lane). Co-expression of SOCS1 or SOCS5 dramatically inhibited JAK1 tyrosine phosphorylation. In comparison, co-expression of SOCS2, SOCS3, SOCS4 or SOCS6 effected a modest inhibition, whilst co-expression of SOCS7 had no effect (Fig. 1A). Although JAK1 is a known SOCS3 target, SOCS3 does not inhibit in this assay because the majority of JAK1 is not associated with receptor complexes. This is consistent with previous experiments [24], [50]. To efficiently inhibit, the SOCS3-SH2 domain needs to be bound to receptor (for example Tyr757 in gp130 [51]) [27].

### SOCS5 can inhibit JAK1 and JAK2, but not JAK3 or TYK2 activation

To investigate whether SOCS5 preferentially inhibited JAK1 activation in this system, 293T cells were transiently transfected with expression vectors encoding Flag epitope-tagged JAK1, JAK2, JAK3, or TYK2 with or without Flag-tagged SOCS1 or SOCS5. Proteins were immunoprecipitated using anti-Flag antibody and JAK phosphorylation assessed using phosphospecific or anti-phosphotyrosine antibodies, as indicated. Co-expression of SOCS5 dramatically inhibited JAK2 (Fig. 1B), but did not inhibit JAK3 or TYK2 phosphorylation (Fig. 1C), indicating a high degree of specificity in regulation of individual JAK family members.

### The N-terminal region is critical for inhibition of JAK1 phosphorylation

To determine which regions of SOCS5 were required for inhibition of JAK1 activation, SOCS5 mutants which lacked either the entire N-terminus (Δ369; a.a. 370–536) or part thereof (Δ110: a.a. 111–536; Δ171: a.a. 172–536; Δ313: a.a. 314–536; Δ349: a.a. 350–536), or contained a mutated SH2 domain (R406K; mSH2) or SOCS box (L484P, C488F; mSB), were generated to express proteins with N-terminal Flag epitopes. We also assessed the functional importance of the region adjacent to the SOCS5-SH2 domain by mutating His360 (H360A; homologous to the critical phenylalanine residue within the SOCS1 and SOCS3 KIR region) [26], [50]. 293T cells were again transfected with the Flag-tagged JAK1 expression plasmid, with and without constructs for expression of the various Flag-tagged SOCS5 mutants (Fig. 1D & E). Mutation of the SH2 domain or SOCS box had a moderate effect on SOCS5 function, resulting in less inhibition of phosphorylated JAK1 than that seen with wild-type SOCS5 (Fig. 1D, upper panel). This was in contrast to deletion of the N-terminal region, which strikingly, resulted in complete loss of inhibition by SOCS5 (Fig. 1D & E, upper panel). The first 110 residues appeared to be dispensable for SOCS5 inhibition of JAK1. In contrast, deletion of the N-terminal 171 amino acids resulted in impaired SOCS5 function and further deletion of either 313, 349 or 369 residues, resulted in an inability to inhibit JAK1 phosphorylation, suggesting that a region between residues 110 to 171 contributes significantly to the inhibition of JAK1 (Fig. 1E, upper panel). The apparent increase in JAK1 phosphorylation in the presence of Δ369 and Δ349 SOCS5 (Fig. 1D) was not consistently observed in replicate experiments. Intriguingly, mutation of His360 in the putative SOCS5 KIR region had only a modest impact on JAK1 activation compared to deletion of the N-terminus (Fig. 1D), indicating that SOCS5 may be affecting JAK1 phosphorylation via a novel mechanism, distinct from that of SOCS1 and SOCS3. Re-probing with anti-Flag antibodies revealed appropriate levels of immunoprecipitated proteins (Fig. 1A–E, lower panels). To determine whether SOCS5 could interact with full-length JAK, 293T cells were transfected with a Myc-tagged SOCS5 expression construct, with and without constructs for expression of Flag-tagged JAK1, JAK2, JAK3 and TYK2. Anti-Flag immunoprecipitates were then analyzed for JAK-associated SOCS5 by Western blot with anti-SOCS5 antibodies. SOCS5 was clearly detected in the JAK immunoprecipitates, indicating an interaction with all four members of the JAK family (Fig. 1F, top panel). Reprobe of the membranes confirmed the presence of Flag-tagged JAK proteins (Fig. 1F, middle panel), whilst Western blot of the lysates confirmed expression of SOCS5 in all samples (Fig. 1F, bottom panel).

### SOCS5 can directly inhibit JAK1 enzymatic activity

**Figure 2:**
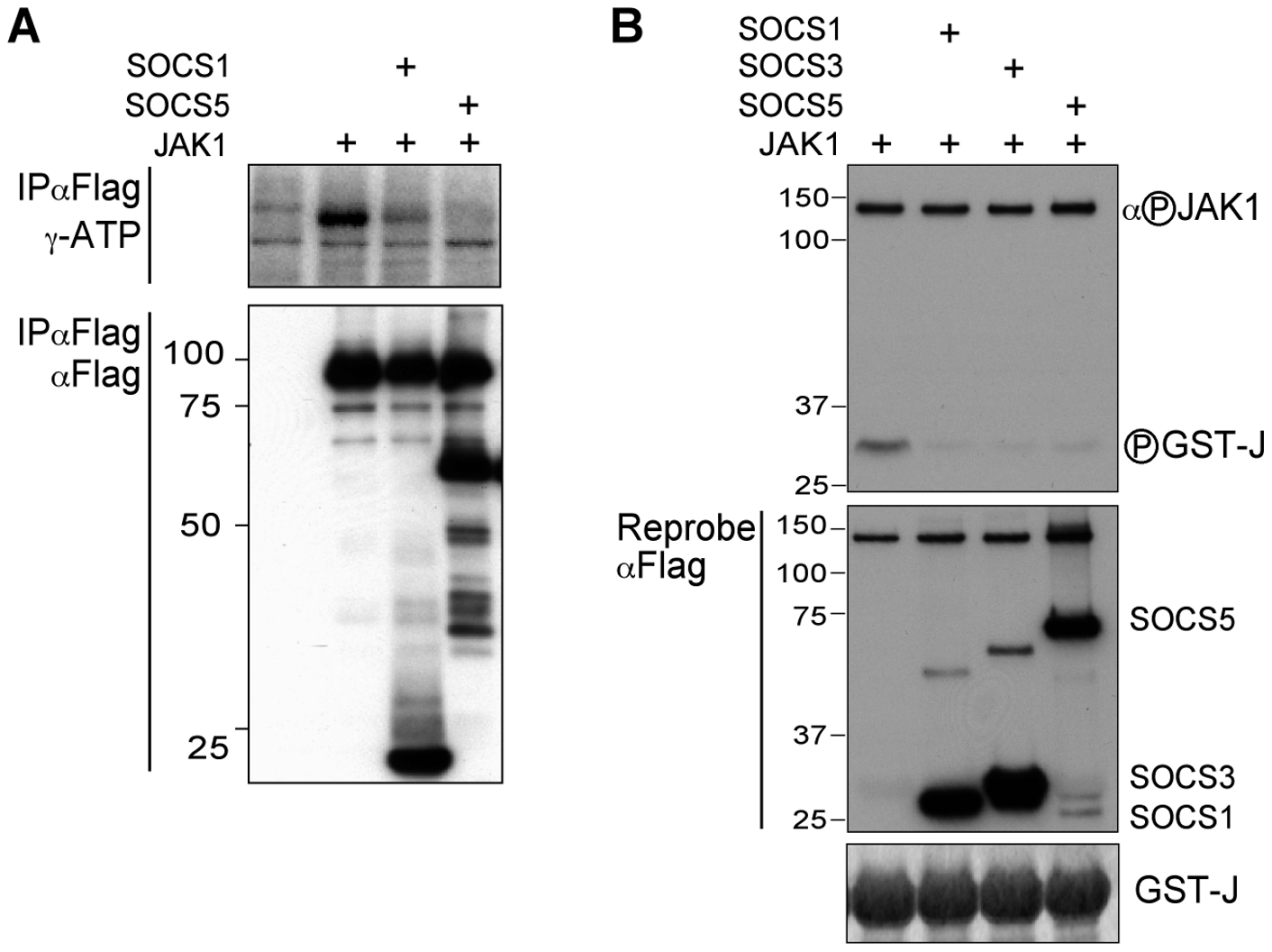
SOCS5 inhibits JAK1 kinase activity. (A) 293T cells were transfected with cDNA encoding Flag-tagged mouse JAK1 (+) in the presence or absence of cDNAs encoding Flag-tagged SOCS1 or SOCS5. Anti-Flag immunoprecipitates were incubated in the presence of ^32^P-γ-ATP at 37°C. Incorporation of ^32^P was visualised by autoradiography (top panel). Immunoprecipitates were analyzed by Western blot with anti-Flag antibodies (lower panel). (B) cDNAs encoding Flag-tagged SOCS1, SOCS3, SOCS5 or JAK1 were independently transfected into 293T cells. Proteins were immunoprecipitated using anti-Flag antibody, and eluted from the resin by competition with Flag peptide. Proteins were then mixed and an *in vitro* kinase assay performed. JAK1 autophosphorylation and phosphorylation of the GST-Jak2 activation peptide (substrate; GST-J) (top panel) were assessed by Western blotting with phospho-specific antibodies. A sample of the reaction mix was analyzed by Coomassie staining to show substrate input (lower panel).

Although SOCS5 could inhibit phosphorylation of Tyr1033 in the JAK1 catalytic loop (Fig. 1A) and phosphorylation of this residue is required for complete enzyme activity, it was not clear whether SOCS5 was directly inhibiting JAK1 catalytic activity. To first confirm that the inhibition of JAK tyrosine phosphorylation (Fig. 1A) reflected loss of JAK enzymatic activity, *in vitro* kinase assays were performed examining JAK autophosphorylation. 293T cells were transiently transfected with constructs encoding Flag-tagged JAK1 and either Flag-tagged SOCS1 or SOCS5, lysed, and the proteins immunoprecipitated using anti-Flag antibodies. Immunoprecipitates were then incubated in the presence of [γ-^32^P]-ATP and phosphate incorporation analyzed. Expression of either SOCS1 or SOCS5 inhibited JAK1 autophosphorylation (Fig. 2A, upper panel), with Western blot analysis of the immunoprecipitates revealing appropriate levels of all proteins (Fig. 2A, lower panel).

To investigate whether SOCS5 could inhibit JAK1 phosphorylation of substrate, 293T cells were transiently transfected with constructs encoding Flag-tagged JAK1 or Flag-tagged SOCS1, SOCS3 or SOCS5, lysed, and the proteins immunoprecipitated using anti-Flag antibodies. JAK and SOCS proteins were eluted using Flag peptide, mixed and incubated in the presence of ATP and a JAK1 substrate (GST-JAK2 peptide [49]). Both SOCS1 and SOCS3 inhibited JAK1 kinase activity as measured by phosphorylation of the substrate using anti-phosphoJAK antibodies, but did not inhibit JAK1 autophosphorylation under these conditions. The SOCS5 inhibition of JAK1 substrate phosphorylation was comparable to that of SOCS3 (Fig. 2B), demonstrating for the first time that SOCS5 can directly inhibit JAK1 activity.

### A conserved N-terminal fragment interacts directly with the JAK JH1 domain

Previous bioinformatic analysis of the N-termini of the SOCS proteins revealed a 70 residue region of high sequence homology present in SOCS4 and SOCS5 (residues 175–244 of mouse SOCS5), which was predicted to contain some secondary structural features [30]. As our functional studies demonstrated that residues between 110–313 were critical for the inhibition of JAK1 activation by SOCS5, we hypothesized that this region might be responsible for these effects. To this end, recombinant protein corresponding to mouse SOCS5^175–244^ was expressed and purified from *E. coli*.

**Figure 3:**
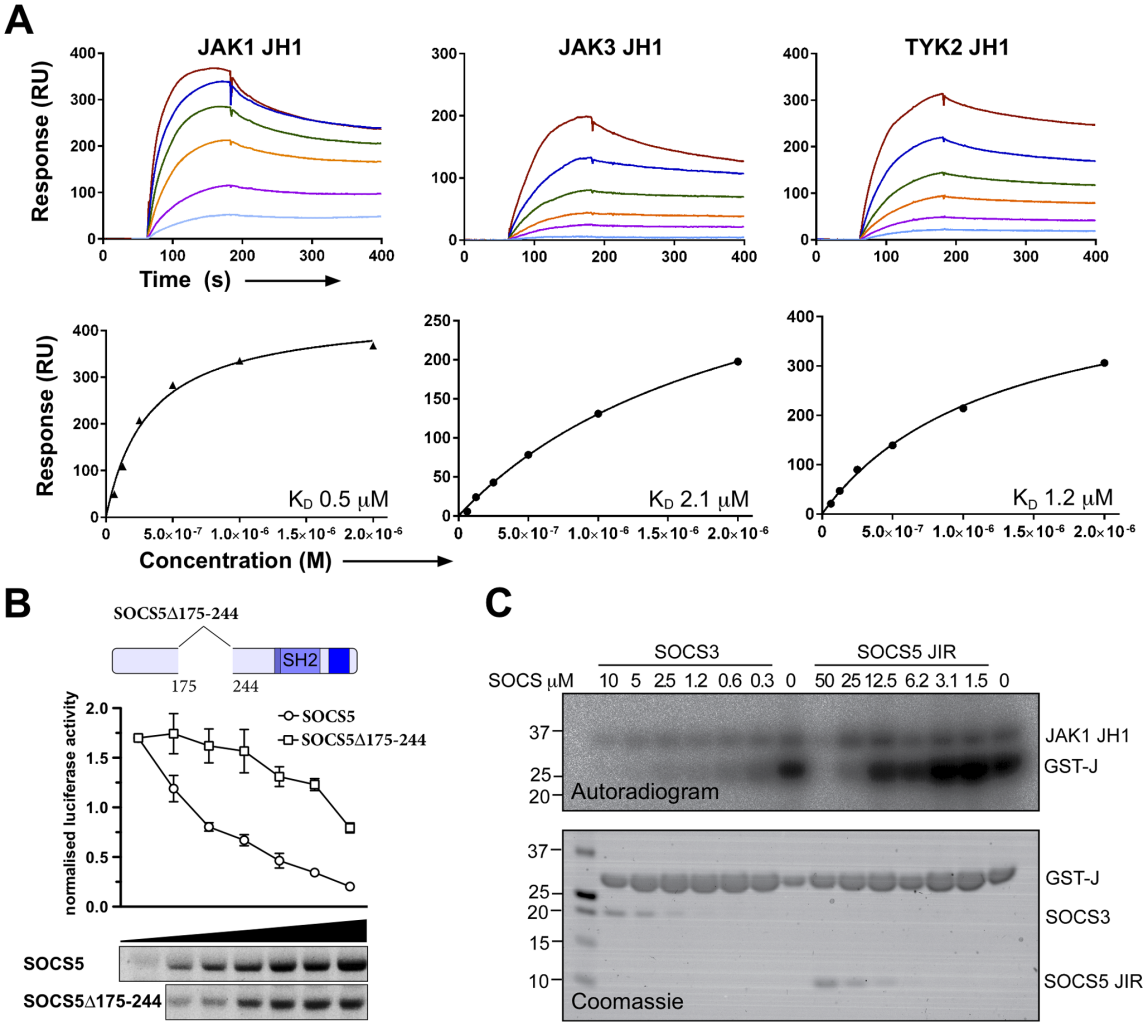
An N-terminal fragment corresponding to residues 175–244 of SOCS5 can directly bind JAK1. (A) SPR analysis of SOCS5^175–244^ fragment binding to the JAK JH1 domain. Serially diluted JAK JH1 domains (62.5 nM–2 μM) were flowed over immobilised SOCS5^175–244^ protein. Upper panels represent sensorgrams showing the kinetics of binding. Lower panels show steady-state analysis. (B) 293T cells were transfected with the Stat6 reporter and increasing amounts of cDNA expressing Flag-tagged SOCS5 (3.13–100 ng) or SOCS5 lacking the conserved N-terminal fragment (9.5–300 ng; Δ175–244) and stimulated overnight with 10 ng/mL rhIL-4. Cells were lysed and induced luciferase activity measured and normalised according to Renilla activity. Data are expressed as arbitrary units and represent the mean of triplicates ± SD. Cell lysates were analyzed by Western blotting for Flag-tagged proteins (SOCS5 upper; Δ175–244 lower panel); images were generated from the same gel and exposure. (C) Recombinant SOCS5 JIR or SOCS3 was incubated with 20 nM JAK1 and GST-JAK2 activation peptide (substrate; GST-J) for 15 min in the presence of 2.5 mM Mg/^32^P-γ-ATP at 37°C. Incorporation of ^32^P was visualised by autoradiography (top panel) and protein input by SDS-PAGE and Coomassie staining (lower panel).

The SOCS5^175–244^ fragment was immobilised by amine coupling to a CM5 biosensor chip and the binding affinity for recombinant JAK1 JH1 domain measured by SPR. The SOCS5^175–244^ fragment bound the JAK1 kinase (JH1) domain with an equilibrium dissociation constant (*K*_D_) of 0.5 μM (Fig. 3A) demonstrating a direct interaction between SOCS5 and the JAK1 JH1 domain. We next tested whether this fragment also bound the JH1 domain of JAK2, JAK3, or TYK2, or the Src kinase domain, and thus might be responsible for the selective inhibition of JAK1 and JAK2 observed in 293T cells (Fig. 1C). SOCS5^175–244^ bound with comparable affinity to the JAK3 and TYK2 JH1 domains (1–2 μM) (Fig. 3). By immobilising the JAK1 and JAK2 JH1 domains on the biosensor chip and comparing binding of the SOCS5^175–244^ fragment by SPR, we also detected binding to the JAK2 JH1 domain (Figure S1 in File S1). Non-specific binding of SOCS5^175–244^ to the reference surface precluded accurate quantitative analysis of the data, resulting in an inability to calculate relative affinities. No binding was observed for the Src kinase domain (data not shown). This indicates that the region corresponding to SOCS5^175–244^ has the potential to bind all four JAK kinases, but an additional region/s of SOCS5 determines the selective inhibition within the JAK family. We therefore propose that the region of the SOCS5 N-terminus encompassing residues 175–244 be termed a JAK interaction region (JIR).

Having established that SOCS5 bound directly to the JAK1 JH1 via its JIR, we next investigated whether this region was functionally important. SOCS5 has previously been shown to inhibit IL-4-induced Stat6 activity [32]. 293T cells were therefore transiently transfected with plasmids expressing Flag-tagged SOCS5 or SOCS5 in which the JIR had been deleted (SOCS5Δ175–244), a Stat6 expression vector and luciferase reporter constructs. Following overnight incubation with IL-4, cells were lysed and luciferase activity measured. Deletion of the JIR from the N-terminus reduced the capacity of SOCS5 to inhibit IL-4-induced Stat6 activity by ∼50%, and in a dose-dependent manner (Fig. 3B), suggesting that this region was functionally important.

As deletion of the first 313 residues of the N-terminus of SOCS5 (which includes the JIR) significantly impaired the inhibitory effect of SOCS5 on JAK1 activity (Fig. 1E) and, as we had shown that SOCS5 could act as a JAK kinase inhibitor, we tested whether the JIR alone might directly inhibit active JAK1 JH1 domain in an *in vitro* kinase assay. In contrast to recombinant SOCS3, the addition of the JIR to the reaction only inhibited JAK1 kinase activity at high levels (25–50 μM; Fig. 3C). This suggests that the JIR alone is unlikely to be a JAK inhibitor. The binding of the JIR to all four JAK JH1 domains, further suggests that the role of the JIR may be to facilitate an interaction with JAK, whilst another region of the SOCS5 N-terminus appears to be required for SOCS5 inhibition of JAK1 or JAK2.

### Binding preferences of the SOCS5-SH2 domain and identification of a high affinity interacting partner: Shc-1

Mutation of the SOCS5-SH2 domain had only a modest effect on JAK1 phosphorylation (Fig. 1D). In addition, we were unable to detect an interaction between the recombinant SOCS5-SH2 domain and active JAK1 JH1 domain by SPR (data not shown), indicating that the SOCS5-SH2 domain is unlikely to directly mediate the interaction with JAK1.

**Figure 4:**
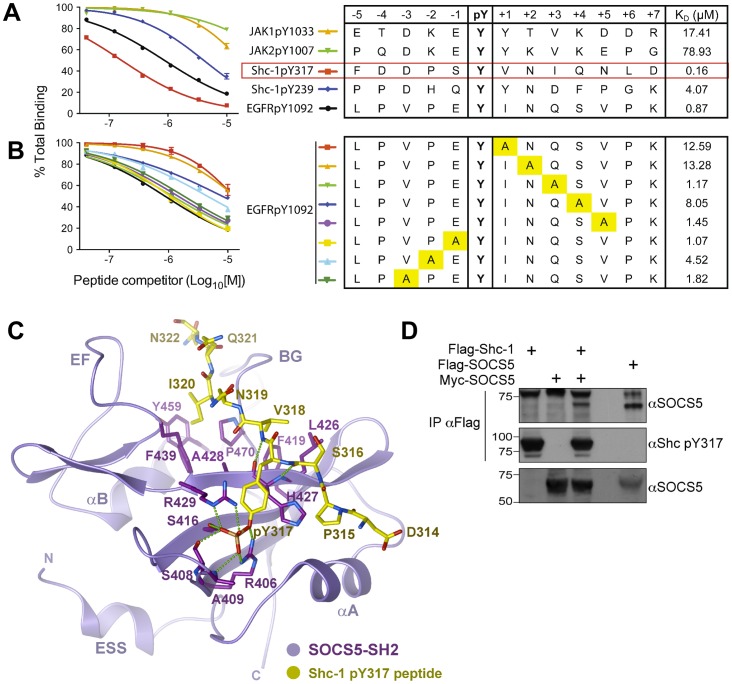
SOCS5-SH2 domain binding analysis and identification of Shc-1 pY317 as a high affinity-potential binding target. SPR analysis of phosphopeptide binding to the SOCS5-SH2 domain. A constant amount of recombinant SOCS5 was mixed with serially diluted phosphopeptides (0.4–10 µM) and flowed over immobilised Shc-1 pY317 peptide. The response units are expressed as a percentage of maximal binding in the absence of competitor and are plotted against the concentration of competitor peptide. Steady-state analysis at saturation of binding was used to derive the *K*_D_ values for the respective phosphopeptides. Binding analysis of (**A**) JAK, Shc-1, or wild-type and (**B**) mutated EGF-R phosphopeptides. Phosphopeptide sequences and the respective *K*_D_ values are shown in the right-hand side table. Yellow boxes highlight residues replaced by an alanine residue. (**C**) Structural model of the SOCS5-SH2-Shc-1 peptide complex. A homology model for the SOCS5-SH2 domain was built using the SOCS4 crystal structure as a template (PDB code 2IZV). The Shc-1 pY317 peptide was modelled from the SOCS3-gp130 crystal structure (PDB code 2HMH). Side chains were optimized using ICM-PRO (Molsoft). The backbone of the flexible EF and BG loops was fixed in the apo-SOCS4 conformation, but is likely to adjust on peptide binding to maximize interactions. Predicted hydrogen bonds are shown as dashed lines. (**D**) SOCS5 interacts with full-length Shc-1 protein. 293T cells were transfected with cDNA encoding Myc-tagged SOCS5 (+) in the presence (+) or absence of cDNA encoding Flag-tagged Shc-1 or alternatively, with cDNA encoding Flag-tagged SOCS5 alone. Cells were treated with 10 μM MG132 for 3.5 h prior to treatment with sodium pervanadate solution for 30 min. Cells were then lysed and anti-Flag immunoprecipitates analyzed by Western blot with anti-SOCS5 antibodies (αSOCS5). The blots were stripped and reprobed with a phospho-specific antibody for Shc-1-Y317 (middle panel). Cell lysates were analyzed by Western blot with anti-SOCS5 (lower panel).

The SOCS4 and SOCS5-SH2 domains share over 92% amino acid sequence homology, suggesting a potential functional overlap in substrate binding. As a first step towards identifying the relevant SOCS4 or SOCS5-SH2 domain interacting partner(s), a complex consisting of GST-SOCS4-SH2 and SOCS box coupled with elongins B and C, was used as bait to affinity-purify proteins from EL4 cell lysates treated with pervanadate and MG132, followed by on-column tryptic digest and Orbitrap LC-MS/MS analysis (Text S1 in File S1). A mutated SOCS4-SH2 domain in which the invariant arginine was replaced with lysine (R308K) was used to distinguish phosphorylation-dependent interactions. Several candidates were identified, including the adaptor protein, Shc-1 (Table S1 in File S1). Shc-1 interacts with multiple growth factor receptors, most notably the EGF-R, and contains well-defined phosphorylation sites (Y239, Y240, Y317) which mediate the recruitment of signaling proteins such as Grb2 [52]–[55]. Previous work had indicated that the related SOCS4-SH2 domain had a strong preference for hydrophobic residues in the +1 and +3 position and bound tightly to EGF-R pY1092 (K_D_ 0.5 µM) [38]. Analysis of the residues flanking the known Shc-1 phosphorylation sites suggested that phosphoTyr317 was a potential binding site, with a sequence related to EGF-R pY1092 (Fig. 4).

Shc-1 pY317 peptide was immobilised and a competitive SPR binding assay established to test binding to GST-SOCS5-SH2 Elo B/C. The Shc-1 pY317 phosphopeptide bound the SOCS5-SH2 domain with a *K*_D_ of 0.16 μM, a 5-fold tighter interaction than that of the EGF-R pY1092 peptide and a 25-fold tighter interaction than for the second Grb2 site on Shc-1 (pY239) (Fig. 4A & B). Binding affinities were also determined for phosphopeptides corresponding to the JAK1(pY1033) and JAK2(pY1007) catalytic loop tyrosines (*K*_D_ 17 and 78 µM, respectively) (Fig. 4A); the relatively low affinities indicate that these sites are unlikely to represent physiological targets of the SOCS5-SH2 domain.

We then investigated the binding preferences for the SOCS5-SH2 domain, utilising the known phosphopeptide ligand for the SOCS4-SH2 domain (EGF-R pY1092) [38] to determine the relative contributions of the flanking residues. Shc-1 pY317 peptide was immobilised and the SPR binding assay used to compare SOCS5 binding to wild-type EGF-R pY1092 and phosphopeptides containing alanine substitutions of the flanking residues. SOCS5 bound the wild-type EGF-R pY1092 peptide with a *K*_D_ of 0.87 µM (Fig. 4B), comparable to that of the SOCS4-SH2 domain [38]. Mutation of isoleucine in the +1, asparagine in the +2 or serine in the +4 position resulted in a reduction in binding affinity. Mutation of proline in the −2 position also resulted in a loss of affinity (Fig. 4B), indicating that the SOCS5-SH2 domain (like other SOCS SH2 domains) [38], [39], [56] may have an extended binding interface with phosphorylated peptides.

To explore the binding interface on the SOCS5-SH2 domain, it was modelled in complex with the Shc-1 Tyr317 phosphopeptide. The highly related SOCS4-SH2 domain structure [38] was used as a template for the SOCS5-SH2 domain, whilst the conformation of the Y317 phosphopeptide was based on the linear binding of the gp130 Tyr757 phosphopeptide to the SOCS3-SH2 domain [39] (Fig. 4C). The decision to represent the Shc-1 Tyr317 phosphopeptide in a linear configuration (rather than the hairpin formed upon binding of the Shc-1 phosphopeptide to the Grb2-SH2 domain [57]) is based upon the likelihood that a hairpin configuration would result in limited contact with the SOCS5-SH2 residues (Figure S2 in File S1). The homology model predicts that the phosphotyrosyl residue will make contacts with the invariant Arg406, in addition to Ser408, Ala409, Ser416 and Arg429 in SOCS5. Shc-1 Val318 (+1 position) is predicted to form a hydrogen bond with His427 in SOCS5 as well as hydrophobic contacts with Phe419 and Leu426. Shc-1 Ile320 (+3 position) is predicted to occupy a hydrophobic pocket between SOCS5 Phe439, Tyr459 and Pro470 (Fig. 4C).

To confirm that SOCS5 interacts with full-length Shc-1 protein, 293T cells were transiently transfected with expression vectors encoding Myc epitope-tagged SOCS5 in the presence or absence of Flag-tagged Shc-1 or Flag-tagged SOCS5 alone. Cells were treated with MG132 for 3 h to inhibit the proteasome, and sodium pervanadate for 30 min to inhibit phosphatase action and ensure that Tyr317 in Shc-1 was phosphorylated. Cells were lysed and proteins immunoprecipitated using anti-Flag antibody, followed by Western blot with anti-SOCS5 antibody. SOCS5 was specifically associated with Shc-1 immunoprecipitates; whilst Shc-1 phosphorylation was confirmed by reprobe of anti-Flag immunoprecipitates with a phospho-specific antibody for Shc-1-Tyr317 (Fig. 4D).

Collectively, these results reveal a potential new mechanism by which SOCS5 may play a role in regulating Ras/MAPK signaling, not only in the context of EGF and growth factor signaling, but also in the context of increased phosphorylation of Shc-1, as occurs during oncogenic signaling.

## Discussion

Very little is known regarding the signaling cascades regulated by SOCS4 and SOCS5, and while both JAK and the EGF-R have been suggested as potential targets, our understanding of the biochemical mechanism/s of action employed by these two proteins is limited, and largely inferred from our knowledge of other SOCS family members. Here, we have shown using co-expression in 293T cells that while SOCS5 can specifically interact with all four JAKs it selectively inhibits the autophosphorylation of JAK1 and JAK2. The interaction is likely to be mediated by the identified, conserved JAK interacting region (JIR) in the SOCS5 N-terminus, whilst the inhibition appears to require an additional region within the SOCS5 N-terminus. Given that by homology, the JIR is also present in the SOCS4 N-terminus [30], this leads us to speculate that the physiological roles of these two orphan SOCS proteins will involve regulation of JAK kinase function. However, the modest inhibition of JAK1 phosphorylation by SOCS4 (when compared to SOCS1 and SOCS5; Fig. 1A) suggests that although the conserved region or JIR in SOCS4 may be able to bind to JAK1, the two proteins will be functionally distinct. Further experiments are needed to address the functional role of the SOCS4 JIR.

**Figure 5:**
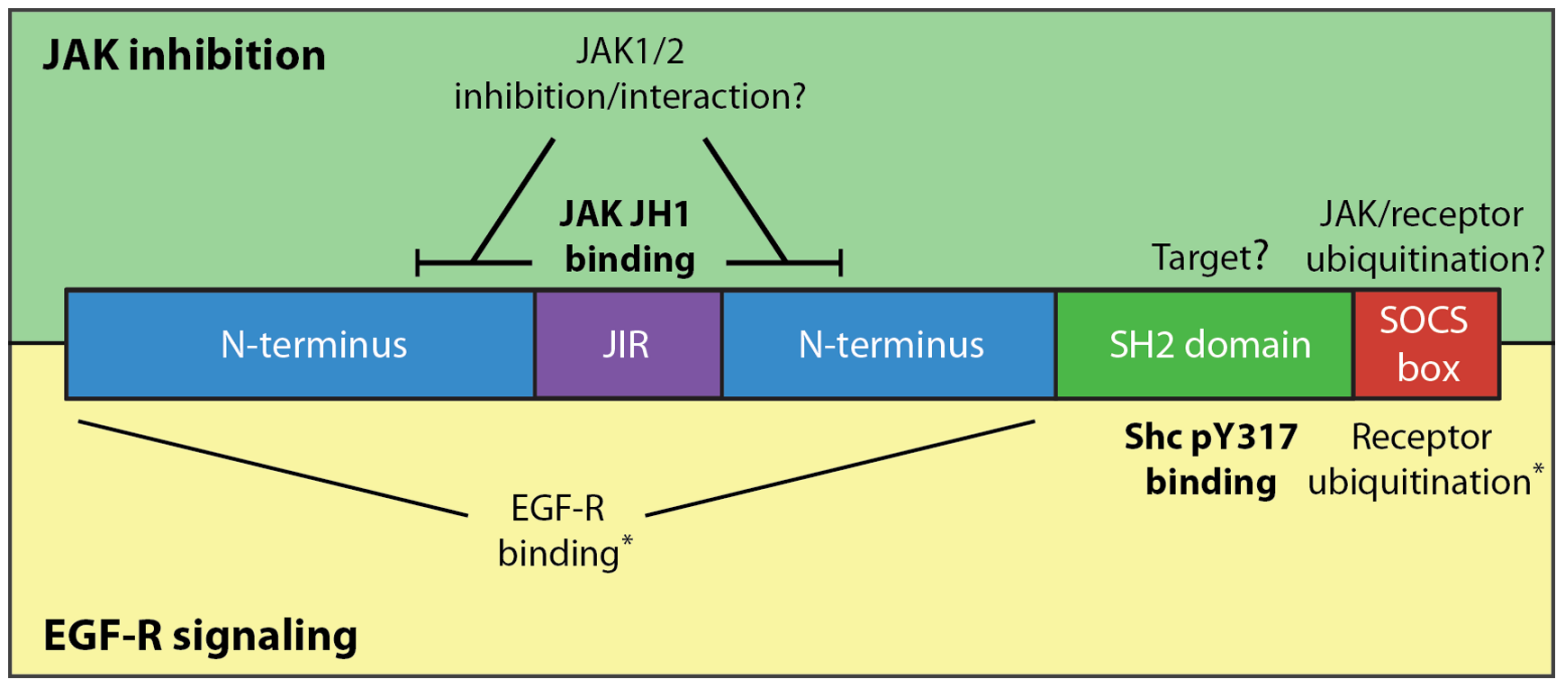
Distinct domains within SOCS5 mediate interaction with JAK and Shc. Schematic showing SOCS5 domain organization and the regions implicated in JAK interaction and inhibition (upper section) in comparison to those involved in inhibition of EGF-R signaling and binding to Shc-1 (lower section). *indicates regions of SOCS5 previously reported to be involved in interaction and degradation of the EGF-R (N-terminus and SOCS box, respectively) [7], [8]. JIR: JAK Interaction Region.

While caveats must be applied to observations obtained using overexpressed proteins, our results revealed a striking specificity in the ability of SOCS5 to regulate JAK, with selective inhibition of JAK1 and JAK2, but not JAK3 or TYK2 phosphorylation. Specificity did not appear to be determined by interaction of the SOCS5 JIR with JAK, as this region appeared to bind similarly to the JAK1, JAK2, JAK3 and TYK JH1 domains. Deletion analysis of the SOCS5 N-terminus indicated that additional residues, yet to be defined (for example between residues 110 and 174, or 246 and 313; Fig. 1E), are likely to determine the specificity of inhibition by SOCS5. The additional residues may contribute to either inhibition of JAK activity or provide a tighter binding interaction with JAK1 and JAK2 (summarised schematically in Fig. 5). As the sequences flanking the JIR do not appear to be highly conserved between SOCS4 and SOCS5 [30], this may also explain the inability of SOCS4 to inhibit JAK1 (Fig. 1A).

Interestingly, although SOCS5 was able to inhibit JAK1 and JAK2 autophosphorylation when co-expressed with JAK (Fig. 1A & B and Fig. 2A), it was unable to inhibit JAK1 autophosphorylation in the *in vitro* kinase assay (Fig. 2B). When JAK1 and SOCS5 are co-expressed in cells, JAK1 is continually being phosphorylated and de-phosphorylated during the course of the transfection, and SOCS5 presumably interacts with active (phosphorylated) JAK1 to inhibit further enzymatic activity; the net result of which is inhibition of autophosphorylation. In the *in vitro* kinase assay (Fig. 2B), full-length JAK1 and SOCS5 are produced independently, so that JAK is active at the start of the assay. Here we addressed whether SOCS5 could inhibit phosphorylation of a substrate (in the presence of phosphatase inhibitors). In the latter assay, we assume that increased autophosphorylation of active JAK is limiting, in contrast to the phosphorylation of substrate, which is present in excess and therefore provides a much greater dynamic range. We cannot exclude a contribution by the SOCS box associated E3 ligase when SOCS5 and JAK are co-expressed in cells (Fig. 1D).

Although the ability of full-length SOCS5 to inhibit JAK enzymatic activity was comparable to that of SOCS1 or SOCS3 (Fig. 1F), it seems likely that the mechanism of inhibition will be distinct from these two well-characterised JAK inhibitors. SOCS5 clearly requires at least two regions in the N-terminus (JIR, and an additional region) plus the SH2 domain, for full inhibition of JAK1 (Fig. 1D & E). SOCS1 and SOCS3 interfere directly with JAK kinase activity via their KIR. In contrast, mutation of His360 in the analogous region of SOCS5 had little effect on inhibition of JAK1 phosphorylation (Fig. 1D). Additionally, a chimera of SOCS3, in which the KIR was replaced by the equivalent SOCS5 region, did not inhibit JAK2 kinase activity *in vitro* [26]. Similarly, mutation of the SOCS box had only a modest effect on inhibition by SOCS5 (Fig. 1D), suggesting that although ubiquitination and proteasomal degradation may contribute, it is not the primary mechanism of inhibition, at least not when SOCS5 is expressed at high levels in 293T cells.

While the SH2 domain appeared to have a minor role (relative to the N-terminus) in the SOCS5 inhibition of JAK phosphorylation, it is likely to have a more important role in a physiological setting. Prior to this study, no substrates had been identified for the SOCS5-SH2 domain. Our preliminary peptide binding analysis suggests a preferred consensus of “P X pY Φ N Φ S” where X denotes any residue, and Φ denotes any hydrophobic residue, and enables candidate binding targets to be interrogated for SOCS5 substrate sequences. We note that neither the JAK1 nor JAK2 JH1 domain contains a sequence corresponding to this consensus. Our studies have identified Shc-1 as a novel candidate for regulation by SOCS5. The measured binding affinity of the SOCS5-SH2 domain for Tyr317 in Shc-1 (0.16 μM, Fig. 4A), is comparable to that observed between SOCS3 and its physiological ligand, Tyr757 in gp130 (0.1–0.15 μM [40], [51]) and suggests that phosphorylated Tyr317 on Shc-1 is likely to represent a biologically relevant target.

EGF activation of the Ras-mitogen activated protein kinase (MAPK) pathway occurs through the recruitment of Grb2 and Shc-1 to tyrosines within the EGF-R cytoplasmic domain [52]. Phosphorylation of Shc-1 on Tyr239 and 317 also results in the recruitment of Grb2 to Shc-1 [55], [58], [59], which then mediates activation of Ras and the downstream MAP kinases. Interestingly, Tyr1138, the Shc-1/Grb2 binding site within the EGF-R intracellular domain (PEYLNTVQ), along with Tyr1092, are potential SOCS5 binding sites. Identification of Shc-1 pTyr317 as a substrate of the SOCS5-SH2 domain predicts that if SOCS5 expression is increased it could potentially compete with Grb2 for binding to both the EGF-R and Shc-1, thus inhibiting downstream Ras/MAPK signaling.

Consistent with their high sequence homology, the SOCS4 and SOCS5-SH2 domains bind with comparable affinity to the Shc-1 Tyr317 phosphopeptide (data not shown), suggesting that these proteins might be functionally redundant in their ability to regulate Shc-1 pathways. The role of the SOCS5 N-terminus remains unclear in this context, although our previous work suggests that the N-terminus is required for recruitment to the EGF receptor complex prior to ligand stimulation [8].

The SOCS5 interaction with Shc-1 is likely to have wider consequences than regulation of EGF signaling. Shc-1 is involved in transducing signals from many tyrosine kinase receptors, such as the insulin receptor, c-Met and M-CSF receptor [60]–[62], as well as from receptors that utilise the JAK kinases, such as GM-CSF and IL-3 [63], and from the antigen receptors in T and B lymphocytes [64]. While SOCS5 appears to be widely expressed at a tissue level, identification of the inducing stimuli and a careful analysis of the cellular subsets in which it is expressed will be required to fully understand its biological role. This is most pertinent to the question of functional redundancy between SOCS4 and SOCS5, including whether these two SOCS proteins are differentially regulated in response to cytokines and growth factors.

Although preliminary, our data show that via specific regions within its N-terminal region, SOCS5 has the potential to regulate JAK1 or JAK2 activity, while both SOCS4 and SOCS5 may retain the ability to regulate Shc-1-mediated signaling through binding of their SH2 domains to Tyr317. In conclusion, this study identifies two distinct mechanisms by which SOCS5 can regulate cytokine and growth factor signaling, and positions SOCS5 as a potential regulator of multiple growth and chemotactic stimuli, many of which are pivotal to cellular transformation and metastatic disease. Future work will address the significance of these observations in animal models of tumorigenesis.

## Supporting Information

File S1 **Suppressor of cytokine signaling (SOCS) 5 utilises distinct domains for regulation of JAK1 and interaction with the adaptor protein Shc-1.**
(DOCX)
