# Supplementary material for: Suppressor of Cytokine Signaling (SOCS) 5 Utilises Distinct Domains for Regulation of JAK1 and Interaction with the Adaptor Protein Shc-1
Source: PLoS One. 2013 Aug 21;8(8):e70536. doi: 10.1371/journal.pone.0070536 (PMC3749136; doi:10.1371/journal.pone.0070536)
Supplement: File S1 — Suppressor of cytokine signaling (SOCS) 5 utilises distinct domains for regulation of JAK1 and interaction with the adaptor protein Shc-1. (DOCX) [file pone.0070536.s001.docx]

**Supplementary data**

**Suppressor of cytokine signaling (SOCS) 5 utilises distinct domains for regulation of JAK1 and interaction with the adaptor protein Shc-1**

Edmond M. Linossi^1,2^, Indu R. Chandrashekaran^3^, Tatiana B. Kolesnik^1,2^, James M. Murphy^1,2^, Andrew I. Webb^1,2^, Tracy A. Willson^1,2^, Lukasz Kedzierski^1,2^, Alex N. Bullock^4^, Jeffrey J. Babon^1,2^, Raymond S. Norton^3^, Nicos A. Nicola^1,2^, Sandra E. Nicholson^1,2^*

*^1^Walter and Eliza Hall Institute of Medical Research, Parkville, Victoria, Australia; ^2^The University of Melbourne, Parkville, Victoria, Australia; ^3^Monash Institute of Pharmaceutical Sciences, Monash University, Parkville, Victoria, Australia; ^4^University of Oxford, Structural Genomics Consortium, Old Road Campus Research Building, Oxford, United Kingdom.*





**Figure S1**. **SPR analysis of SOCS5^175-244^ fragment binding to the JAK2 JH1 domain.** Serially diluted SOCS5^175-244^ (31.2 nM-500 nM) was flowed over immobilised JAK2 and JAK1 JH1 domains. Upper panels represent sensorgrams showing the kinetics of binding. Lower panels show steady-state analysis.

**Text S1. Identification of candidate SH2-binding proteins**

A ternary complex of GST-fused SOCS4-SH2 domain^271-434^ with elongins B and C was used as bait to affinity-purify interacting proteins from the EL-4 T cell lymphoma line. SOCS4-SH2 domain in which the invariant arginine was mutated to lysine (R308K), GST-SOCS3-SH2 domain with elongins B and C [[1](#_ENREF_1)], and GST alone, were used to identify non-specific binders.

Recombinant SOCS4-SH2 domain proteins were engineered to contain an N-terminal GST-tag and included the SOCS box sequences for increased stability and solubility when expressed as a ternary complex with elongins B and C, as previously described [[2](#_ENREF_2)]. *E. coli* expression vectors encoding murine SOCS3 [[1](#_ENREF_1)] or human SOCS4 (residues 274-437; vector pGTvL1-SGC) and elongin B/elongin C (residues 1-118 and 17-112 respectively; vector pACYCDUET) were co-transformed into BL21(DE3) cells for expression and purification of the trimeric SOCS-SH2-SOCS box-elongin B/elongin C complex.

Cells were pre-treated with the proteasome inhibitor MG-132 (4 h; Sigma) and sodium pervanadate (30 min) to enrich for phosphorylated proteins. 1x10^8^ cells were lysed in 1% NP-40 lysis buffer (1% v/v NP-40, 10% v/v glycerol, 20 mM Tris-HCl, pH 7.5, 150 mM NaCl, 1 mM EGTA, 10 mM NaPPi, 5 mM NaF, 1 mM Na_3_VO_4_) containing protease inhibitors and pre-cleared for 1-2 h with glutathione-Sepharose. Clarified lysates were then incubated with 10-20 μg bait protein overnight and protein complexes recovered using glutathione-Sepharose, eluted with 0.16% (v/v) phosphoric acid, pH 1.8, and subjected to a solid-phase tryptic digest, essentially as described [[3](#_ENREF_3)]. Samples were analysed by liquid chromatography and tandem mass spectrometry (LC-MS/MS) on an LTQ-Orbitrap (Thermo Scientific). Acquired files were converted using Mascot Daemon (Matrix Science) and searched against the Swissprot sequence database. Peptide identifications were accepted with a Mascot score of ≥90%. Protein identifications were accepted if at least 3 unique peptides were identified.

A protein was considered as a potential interacting protein if it satisfied the following criteria; absent from all control experiments and detected in 3 or more of the 5 experiments performed. Cullin5 and Rbx2, two known components of the SOCS box associated E3 ligase, were identified by this approach. The adapter protein Shc-1 satisfied the above criteria and summary data corresponding to the number of unique peptides detected are shown in Table S1. Data are also shown for the relevant bait proteins (Table S1, in File S1).

**Table S1. Summary of peptide data identifying Shc-1 as a candidate binding protein.**

| **Identified Proteins** | **MW**  **(kDa)** | **Number of unique peptides** | | | | |
| --- | --- | --- | --- | --- | --- | --- |
|  |  | **1*** | **2** | **3** | **4** | **5** |
| human SOCS4-SH2-SOCS box^1^ | 19 | 10 | 6 | 12 | 13 | 11 |
| human elongin B^1^ | 13 | 22 | 19 | 21 | 18 | 22 |
| human elongin C^1^ | 11 | 9 | 8 | 7 | 6 | 8 |
| mouse Cullin5 (Q9D5V5) ^2^ | 91 | 24 | 29 | 12 | 15 | 21 |
| mouse Rbx2 (Q9WTZ1)^2^ | 13 | 3 | 3 | 3 | 0 | 3 |
| mouse Shc-1 (P98083) | 63 | 6 | 9 | 0 | 0 | 3 |

*1-5 represent data from five experiments.

^1^Bait proteins: SOCS4, elongin B and elongin C.

^2^SOCS box-associated proteins: Cullin5, Rbx2. Accession numbers are shown in brackets.

**
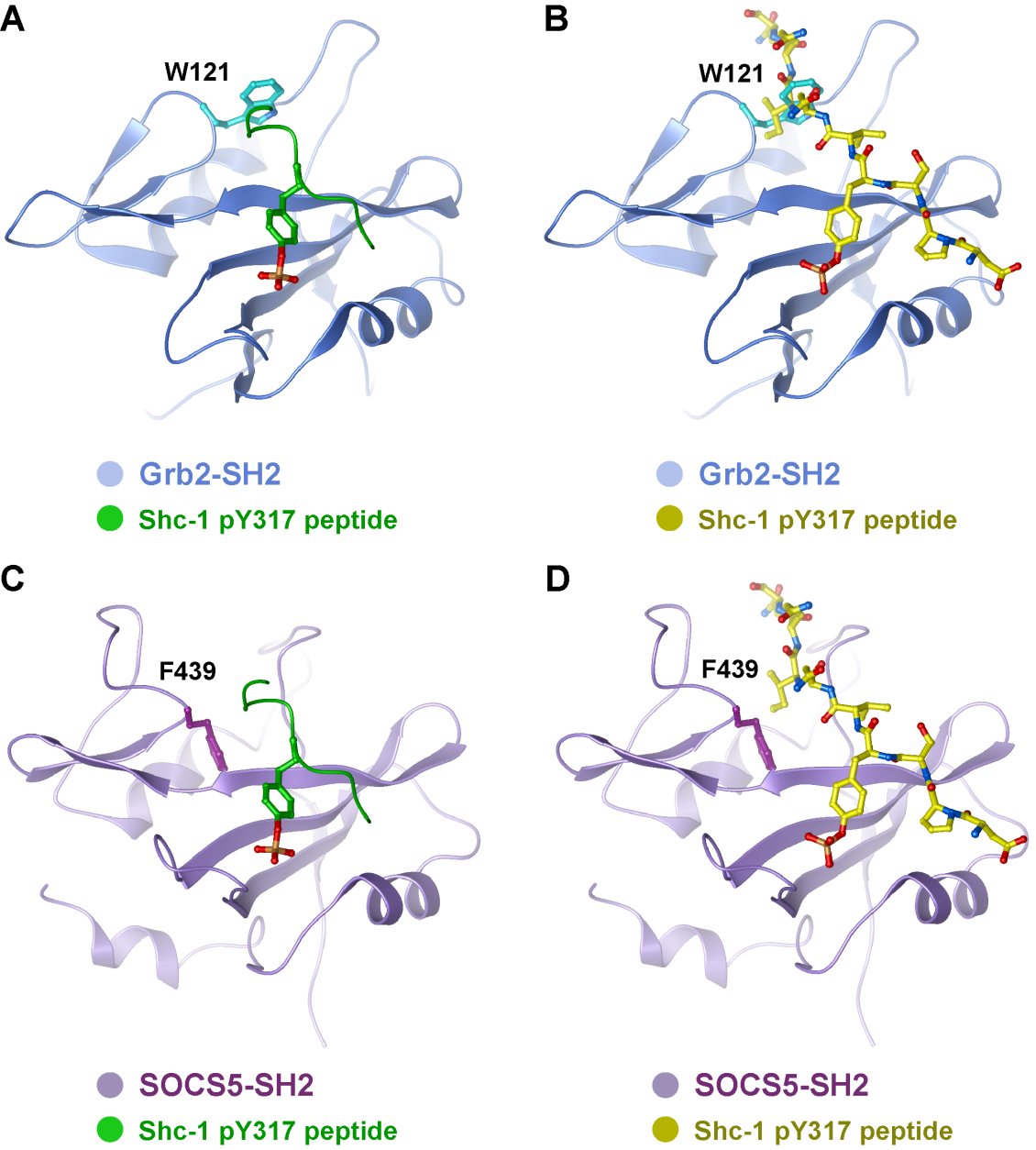
**

**Figure S2. Comparison of the SOCS5 and Grb2 SH2 domains. (A)** In the solution structure of the Grb2-Shc-1 complex [[4](#_ENREF_4)](PDB 1QG1) the bulky Trp121 side chain (Grb2 EF loop) blocks access to the +3 hydrophobic pocket and forces a β-hairpin binding mode for the Shc-1 peptide. **(B)** Modelling of an extended Shc-1 peptide conformation indicates the likely clash with Grb2 Trp121. **(C)** A homology model for the SOCS5-SH2 domain shows the absence of an equivalent Trp residue at this position. The most similar residue is SOCS5 Phe439, which contributes to the +3 hydrophobic pocket. The conformation of this side chain appears not to exclude a β-hairpin (shown) or extended binding mode. **(D)** Model of an extended Shc-1 peptide-binding mode in SOCS5. The peptide shows no clash with Phe349. This configuration is most consistent with the extended peptide-binding mode observed for other SOCS proteins, including the SH2 domains of SOCS3 and SOCS6 [[5](#_ENREF_5),[6](#_ENREF_6)]. In addition, an extended peptide-binding mode is observed for the SHP2-SH2 domain, which binds IRS-1 through a similar pYVNI recognition motif [[7](#_ENREF_7)] (PDB 1AYB).

**References**

1. Babon JJ, Sabo JK, Soetopo A, Yao S, Bailey MF, et al. (2008) The SOCS box domain of SOCS3: structure and interaction with the elonginBC-cullin5 ubiquitin ligase. J Mol Biol 381: 928-940.

2. Bullock AN, Rodriguez MC, Debreczeni JE, Songyang Z, Knapp S (2007) Structure of the SOCS4-ElonginB/C complex reveals a distinct SOCS box interface and the molecular basis for SOCS-dependent EGFR degradation. Structure 15: 1493-1504.

3. Chen C, Jin J, James DA, Adams-Cioaba MA, Park JG, et al. (2009) Mouse Piwi interactome identifies binding mechanism of Tdrkh Tudor domain to arginine methylated Miwi. Proc Natl Acad Sci U S A 106: 20336-20341.

4. Ogura K, Tsuchiya S, Terasawa H, Yuzawa S, Hatanaka H, et al. (1999) Solution structure of the SH2 domain of Grb2 complexed with the Shc-derived phosphotyrosine-containing peptide. J Mol Biol 289: 439-445.

5. Babon JJ, McManus EJ, Yao S, DeSouza DP, Mielke LA, et al. (2006) The structure of SOCS3 reveals the basis of the extended SH2 domain function and identifies an unstructured insertion that regulates stability. Mol Cell 22: 205-216.

6. Zadjali F, Pike AC, Vesterlund M, Sun J, Wu C, et al. (2011) Structural basis for c-KIT inhibition by the suppressor of cytokine signaling 6 (SOCS6) ubiquitin ligase. J Biol Chem 286: 480-490.

7. Lee CH, Kominos D, Jacques S, Margolis B, Schlessinger J, et al. (1994) Crystal structures of peptide complexes of the amino-terminal SH2 domain of the Syp tyrosine phosphatase. Structure 2: 423-438.
